# Learning virulent proteins from integrated query networks

**DOI:** 10.1186/1471-2105-13-321

**Published:** 2012-12-02

**Authors:** Eithon Cadag, Peter Tarczy-Hornoch, Peter J Myler

**Affiliations:** 1 Ayasdi Inc, Palo Alto, CA, USA 94301; 2 Department of Biomedical Informatics and Medical Education, University of Washington, Seattle, WA, USA 98195; 3 Seattle Biomedical Research Institute, Seattle, WA, USA 98109

## Abstract

**Background:**

Methods of weakening and attenuating pathogens’ abilities to infect and propagate in a host, thus allowing the natural immune system to more easily decimate invaders, have gained attention as alternatives to broad-spectrum targeting approaches. The following work describes a technique to identifying proteins involved in virulence by relying on latent information computationally gathered across biological repositories, applicable to both generic and specific virulence categories.

**Results:**

A lightweight method for data integration is used, which links information regarding a protein via a path-based query graph. A method of weighting is then applied to query graphs that can serve as input to various statistical classification methods for discrimination, and the combined usage of both data integration and learning methods are tested against the problem of both generalized and specific virulence function prediction.

**Conclusions:**

This approach improves coverage of functional data over a protein. Moreover, while depending largely on noisy and potentially non-curated data from public sources, we find it outperforms other techniques to identification of general virulence factors and baseline remote homology detection methods for specific virulence categories.

## Background

Though recent decades have seen a decrease in mortality related to infectious disease, new dangers have appeared in the form of emerging and re-emerging pathogens as well as the continuing threat of weaponized infectious agents [1,2], thus creating a strong need to find new methods and targets for treatment. Underscoring the importance of this issue, the National Institute of Allergy and Infectious Disease maintains a categorical ranking of disease-causing microorganisms (NIAID Biodefense Categories) that could cause significant harm and mortality [3]. Broadly, infectious disease remains a global concern and a problem whose impact is most felt in poorer areas of the world. Fortunately, many pathogen genomes have been sequenced and continue to be sequenced, and hold the promise of expediting new therapeutics. As a result, genomic and proteomic sequences are available for many bacterial and viral causes of disease. The National Microbial Pathogen Data Resource (NMPDR), a curated database of pathogen genomes, lists 801 different species and strains bacteria and eukarya infectious to mankind [4]. The availability and dissemination of this data has allowed many new discoveries in virulence research to stem at least partly from computational methods. The challenge is no longer having to work with limited data, but rather how best to exploit the information available and prioritize targets of study.

A critical set of potential genes of interest within a pathogen are those directly involved in pathogenesis. These genes, or virulence factors, can have varying degrees of importance in the initiation and maintenance of infection, and constitute an attractive group of putative targets. Concrete determination of a gene’s involvement in disease is generally left to experimental results, and many studies rely on knockouts or mutations of putative virulence genes [5,6]. Resulting attenuation or avirulence would then be strong evidence that the gene is involved in disease, although the exact function or role may still remain a mystery. Proper target selection is important, however, given that laboratory science makes the identification and verification of virulence factors a costly endeavor. Faster methods are preferred early on that can highlight the most likely targets before experimental assays are carried out.

Identifying and annotating these virulence factors is an early and integral part of understanding how a disease causes damage to a host; improvements in accuracy and speed for finding proteins involved in virulence have the potential to increase the analytical throughput of therapeutic research, provide clues towards mechanisms of infection and provide tempting biomarkers for diagnostic techniques. However, prior computational research in quickly identifying virulence factors has been limited, and has only in the last few years become an area of strong interest for researchers. Several public databases have recently been released that focus exclusively on pathogenesis. Among these include: the Virulence Factor Database (VFDB), a repository of genomic and proteomic data for bacterial human pathogens [7,8]; the Argo database, a collection of virulence factors believed to be involved with resistance for *β*-lactam and vancomycin families of antibiotics [9]; and MvirDB, a aggregated data warehouse of many, smaller virulence-related databases (including VFDB and Argo) [10]. Many of these repositories support standard sequence-based searches against their content, facilitating virulence identification via sequence similarity.

Classification algorithms have also been applied to the problem of virulence recognition for cases where homologies between virulence proteins may be remote. Sachdeva et al., for example, used neural networks to identify adhesins related to virulence [11]. Saha used support vector machines to predict general virulence factors via an approach similar to one proposed in [12] - mapping combinations of the amino acid alphabet to a space such that the presence of a peptide sequence would constitute a classifiable feature [13]. However, the resulting top accuracy for virulence proteins, 62.86%, was relatively low in comparison to the other protein roles predicted (e.g., cellular and metabolic involvement). Work by Garg and Gupta improved on this performance by also relying on polypeptide frequencies in conjunction with PSI-BLAST data in a cascaded support vector machine (SVM) classifier [14]. This approach yielded a higher accuracy of 81.8% and an area under the receiver operating characteristic (ROC) curve of 0.86 for generalized virulence prediction. Other methods have directed attention at specific types of virulence proteins; Sato et al. developed a model for predicting type III (T3) secretory proteins within *Salmonella enterica* and generalized to *Pseudomonas syringae*[15], and McDermott et al. compare multiple computational secretory prediction methods to predict T3/4 effectors on completely novel proteins [16].

The present work expands upon the recent research in computational virulence prediction relying on noisy, weakly-related annotation information rather than direct sequence data, and describes an approach relying on readily available public data for predicting microbial protein relation to virulence. Information from multiple biologic databases are returned wholesale using a retrieval approach that constructs an interlinked graph of connected information, providing broad functional coverage.

Note that the information retrieved may or may not be specifically relevant or correct with regards to the protein of interest; rather than using that information for direct annotation, the aggregate data are used to build a weighted graph used as input to statistical methods trained to recognize virulence proteins. We apply this approach to both overall and specific virulence, and evaluate its performance against competing methods.

## Methods

### Path-based query retrieval

The methodology we adopt relies on retrieving abundant information regarding a protein sequence using a networked query graph. The core of this approach relies on the notion that leveraging multiple sources simultaneously will improve functional coverage of any given query protein.

We describe the basic query and retrieval model here briefly (refer to [17,18] for further details of the logical query model): let a query graph *G* derived from some schema *S* be *G*(*S*) = 〈*V*,*E*〉, where *V * are the nodes and *E* the relations between the nodes. For any concrete instance of a query graph, *V * refers to the individual records returned from any protein sequence query, and *E* the connection between those records (e.g., through external link reference) or the protein sequence directly (e.g., from a pair-wise sequence comparison). *S* constrains *v *∈* V* to specific resources (records of information), and the nature of *e *∈* E *to specific relations between those resources. In this definition, we allow the assignment of weights onto the edges; in the case of pair-wise sequence comparisons, these weights may represent the quality of the alignment between the query protein and other proteins within a resource. Intuitively, a query graph can imagined as a realization of a graph database whose joins are represented by the edges *E*: *v*_*i *_⋈_*d *_*v*_*j*_, where *v*_*i*_,*v*_*j *_∈* V* and *d* is some primary key-like attribute (e.g., RefSeq identifier in a gene record, referencing its product). This concept can be illustrated via a simple BLAST search: a query is *seeded* with a protein sequence, *s*_0_; the results of the query may be *n* other sequences, *s*_1_,…,*s*_*n*_, for which pairwise alignments ((*s*_0_,*s*_*i*_)→*v*_*i*_) are identified.

We extend the notion of a query graph to exploratory query graphs, or a query graphs expanded to the limit of connections defined in *S* such that for any given protein query, all possible connections between records and all records are realized. Following the previous example, all sequences aligned to *s*_0_ may themselves be used as queries against additional databases, generating a larger query graph whose contents may span multiple sources. Exploratory query graphs thus represent a materialization of the entirety of *S* for any given protein sequence query.

Notably, the query and retrieval approach we describe has been employed widely in life sciences research for data management and navigation purposes [19-22]. However, whereas these prior works has focused on the retrieval, curation, and provenance of biological records, this present study is less interested in the quality of each individual datum and instead focuses on the use of the query graphs as a whole to infer (possibly latent) annotations on the initial seeding query that may not be explicitly represented in the query graphs.

### Constructing weighted query graphs from sequence data

We exploit the notion that within a path-based model, the closer a node is to the initial query, the more relevance it likely has to that query, and that those further from the query itself are theoretically of waning relevance. Naturally, the contents of exploratory query graphs will include records that may be quite distant from the initial query. Prior work by others in the field of biologic data representation explored various methods of exploiting the graph structure for inferring the relevance of individual nodes and paths. Bharat and Henzinger [23], for example, describe several algorithms, such as those that use the in- and out-degree of nodes, for the analogous problem of determining topical relevance of hyperlinked documents; Tsuda et al. [24] use a diffusion-based approach to assign weights within protein networks, a method readily adaptable to query graphs; Weston et al. [25] apply a rank propagation algorithm on sequence similarity graphs generated from PSI-BLAST hit values; and Detwiler et al. [26] test a variety of methods, such as Monte Carlo simulations and relevance propagation, to rank nodes in similar graphs. In contrast, our interest is less in the relevance of any individual node, and instead in the value of using the graph globally as a representation of the query for classification activities.

After seeding an initial query, retrieving records and fully expanding the query graph, we transform the contents of the graph into a representation more amenable to classification by weighting nodes in the query graphs and representing their records as numerical features that can be used as inputs to statistical classification methods. This approach bypasses the difficulties in comparing query graphs directly, and depending on the weighting scheme used can still leverage the benefits of the graph structure. Because the query graph is generated by a series of linkages across databases, groups of nodes that share edges most often or have strong sequence-similarity to the query can be weighed highest. An ideal scheme would heavily weigh nodes that characterize the query sequence more precisely, and lightly weigh nodes that do not, thereby minimizing noise.

Begin by letting *w*_*t*_(*n*) be the weight of node *n* in the query graph at some iteration *t*, and that 0 ≤* w*_*t*_(*n*) ≤ 1, for all *t,n*. This represents the grounding that a user’s posed query is the most confident node within the graph, and that further confidences emanating from resultant queries are derivative of this, and propagate outwards. An illustrative way of representing degradation of confidences between nodes in an exploratory, query sequence-based graph would be expect values from BLAST-based alignments; let *expect*(*p,n*) represent the expect value from some query *p* to the result *n*. Thus, the influence of a node’s inward-joining neighbors may be represented as a factor of both those neighbors’ weights (*w*_*t*−1_(*p*)) and their relation to the target node. Define *ψ* as some function that map some value between relations (*p*,*n*) ∈* E *such that *ψ*(*p*,*n*) is within the domain of (0,1). We can then represent weights of nodes within the query graph by:

(1) $$w_{t}(n)\leftarrow \lambda \left(1-\underset{(p,n)\in E}{\prod }\left(1-w_{t-1}(p)\psi (p,n)\right)\right),$$

where *λ*→[0,1) is a path degradation rate and serves a similar purpose as the PageRank damping factor [27], representing belief that information further from the initial query is of decreasing relevance. For all experiments described in the remainder of this work, *λ *= 0.7 was used and *ψ* is set to:

(2) $$\psi (p,n)=\left|\frac{{\text{log}}_{10}\left(\text{expect}(p,n)\right)}{300}\right|,$$

the above being an empirically determined from [28]. All nodes were given initial weights of 0, save the query itself, which is given a weight of 1, and the algorithm iterates until convergence. Applying weights in this manner takes into account the notion that some nodes will be more well-connected than others. Consequently, nodes with more incoming edges will have a higher weight than nodes with less, all other things being equal.

### Query subgraphs as features

We were primarily interested in the use of query graphs generated from sequences for use as a “fingerprint” in identifying virulent and non-virulent proteins, so once a graph was weighted it was transformed into a feature representation. Let **v** represent a vector of weights from a single data source (and thus constitute the weights for a subgraph of an entire query graph). We represent any query graph as several feature vectors, depending on the number of sources, and implicitly capture the presumed relevance of the node under the weighting scheme described earlier. Transformation of the graph weights to fit the feature vector space model is straightforward, and missing data treated simply. Given a data source *D* with subset of known records *H* (*H *⊆* D*), the feature vector **v** for *G* on *D* is:

(3) $$v^{T}=\{\forall v\in H:v_{0},\cdots \;,v_{n}\},$$

where,

$$n_{}=\left\{\begin{array}{ll} w(n) & \text{if}\;n\in (H\cap V)\\ 0 & \text{otherwise.} \end{array}\right)$$

In the above, *w*(*n*) may take the value of any arbitrary weighting scheme. If **v** has known classification, it would then be possible to use it as a member instance of a label in classification training.

**Figure 1:**
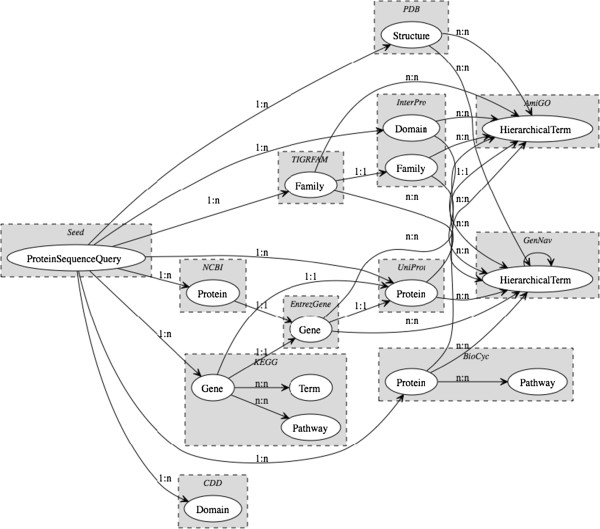
**Integration schema.** Schema used for virulence identification. The records whose weights were converted into features for classification were derived from PDB structures [29], GO terms (from both AmiGO and GenNav) [30,31], InterPro domains and families [32], TIGRFAM families [33], BioCyc pathways [34], KEGG terms and pathways [35], and CDD domains [36]. Note that GenNav is a recursive source - that is, it may re-query itself to recreate the GO hierarchy within the query graph.

The query graphs for the evaluation were generated using the sources and schema shown in Figure 1, and of the sources incorporated into the schema all except for EntrezGene, EntrezProtein and UniProt were tested for classifying abilities. Features upon which classifiers were developed were uniquely identifiable database records. For example, features from AmiGO and GenNav were GO terms across all three ontologies (e.g., ‘GO:0008237’) ; features from CDD were conserved domains (e.g., ‘cd07153’); features from KEGG included pathways (e.g., ‘bme00010’), etc. While two sources represent GO terms (AmiGO, GenNav), there was an important distinction between the two: AmiGO provided only terms directly being referenced by other sources, whereas GenNav additionally provided the ancestors of terms. As a result, feature vectors built from GenNav reconstructed portions of the GO graph within each query instance, allowing us to later compare the utility of discrimination using reference terms versus reference terms within their hierarchical context.

### Datasets for general and specific virulence

The above method and implementation provides a means to query a protein, weight the nodes in the query graph and transform the results into a feature representation suitable for training and classification. We evaluated virulence and non-virulent protein prediction using information derived from query graphs using two different datasets – one for general virulence, and one for specific virulence subcategories.

#### General virulence dataset

We identified a curated set of proteins with which to evaluate the performance of our approach in the form of the non-redundant protein set used by Garg and Gupta to test their own virulence detection system [14], and adopt their train-test procedure to allow direct comparison. Though composed of virulence proteins with a variety of functions, Garg and Gupta treated the entire set as ‘general virulence’ test cases. The positive, virulent number of examples in the set was 1025, with 820 of these acting as training instances for cross-validation and parameter selection, and the remaining 205 for testing. Likewise, the non-virulent proteins numbered 1030, with a division of 206 and 824 for testing and cross-validation, respectively. This constituted an 80%-20% train-test split, with the larger fraction used to optimize the parameters for each algorithm and the smaller used for final testing.

#### Specific virulence dataset

The dataset developed by Garg and Gupta lacked the annotation granularity needed to determine specific virulence roles a protein may play, since the dataset was purely binary in classification, and a protein was categorized as either ‘virulent’ or ‘non-virulent’. For a more specific prediction of virulence factors, we relied on a data warehouse of virulence proteins mentioned earlier, MvirDB. In order to transform the protein data in MvirDB into a suitable training and testing set, the first step was curation of the data into a non-redundant, representative set of proteins.

The original MvirDB dataset consisted of 14544 records. The few DNA sequences in this set were translated to protein sequences, beginning at the leading methionine if present, using the longest open reading frame; otherwise, the DNA sequence was removed from the set. Databases whose contents were viral sequences were removed from the set. These initial filters yielded 5052 remaining proteins.

For negative training and test instances, 3000 proteins were randomly drawn from GenBank [37] and filtered for proteins highly likely to be involved in virulence based on regular expression searches on the protein names and annotations. For example, proteins whose names contained ‘drug’ or ‘toxin’ were removed. Proteins from known pathogen organisms were otherwise left undisturbed in the negative set under the notion that not all proteins within an infectious organism are involved in virulence. At the same time, hypothetical proteins whose functions were unknown were also removed from the negative set. Finally, CD-HIT [38] was used to generate non-redundant protein clusters for the positive and negative sets combined, at 40% sequence identity. This last non-redundancy step ensured that proteins used for the evaluation would be dissimilar overall, and permit validation of discrimination in cases of remote homology [39,40]. The final sequence dataset consisted of 3700 proteins, 1703 of which constituted the negative (non-virulent) set and 1997 of which formed the positive (virulent) classes (see Additional files 1 and 2).

Once the datasets were curated for non-redundancy, and possible virulence factors in the case of negative set, the positive set proteins were labeled with specific virulence functions. Labeling was done based on the information regarding the protein readily available from the originating virulence data sources; many of the databases that MvirDB integrated used a native classification system. Virulence proteins were annotated manually, based on the original classifications and literature references of the native databases.

To illustrate the need for manual annotation over the positive dataset, many databases whose focus is on a specific type or family of proteins, such as in the case of Argo and antibiotic resistance proteins, simply annotate all proteins as a single type. As a result, a small number of categories have very many instances. In other cases, annotations appeared idiosyncratic at the deepest level, but may have been subsumed by higher-level annotations. In this regard, the problem faced is similar to that encountered by the curators of the Unified Medical Language System (UMLS), the Foundational Model of Anatomy (FMA) and GO [41-43] and similarly a solution based on manual comparisons of the various databases’ classifications schemes is used here. This manual annotation process is outlined stepwise in Table 1.

**Table 1:** Procedure for manual curation of virulence factors

	***Procedure for manual curation of virulence factors***
1.	Examine the source or database of each protein annotation for possible classifications, using the annotation set across
	all databases as a starting point. Record annotations according to information from the source or database; each protein
	may have more than one annotation. If a protein is directly involved in a virulence process or *is a regulator* of that process,
	record it as such. In this way, proteins may have more than one annotation.
2.	Examine any publications which are *linked from the source*. Record annotations according to information from the
	publication regarding the protein.
3.	If an annotation was unclear or unknown, conduct a *keyword publication search* of the virulence factor to obtain resolution.
4.	Repeat steps (1-3) across all proteins (i.e. re-annotate) until no further changes were made from the previous annotation.

Iterative method used to manually align and annotate the virulence classifications for virulent proteins in the training and testing dataset.

Manual annotation of the virulence proteins was an iterative process that continued until no further label changes were made to the dataset (either added, changed or deleted). As a result of the manual annotation, 11 top-level virulence-related labels were derived (see Table 2).

**Table 2:** Virulence categories

***No.***	***Virulence category***	***Instance count***
1	Adherence	360
2	Surface factor	66
3	Invasion	249
4	Transport and uptake	225
5	Toxin	319
6	Catalysis	84
7	Secretion	483
8	Motility	181
9	Antibiotic resistance	239
10	Defense	488
11	Other	214

The 11 main virulence categories derived manually from the virulent protein data sources with the number of training and testing records, after 40% identity pruning.

### General virulence prediction evaluation procedure

**Figure 2:**
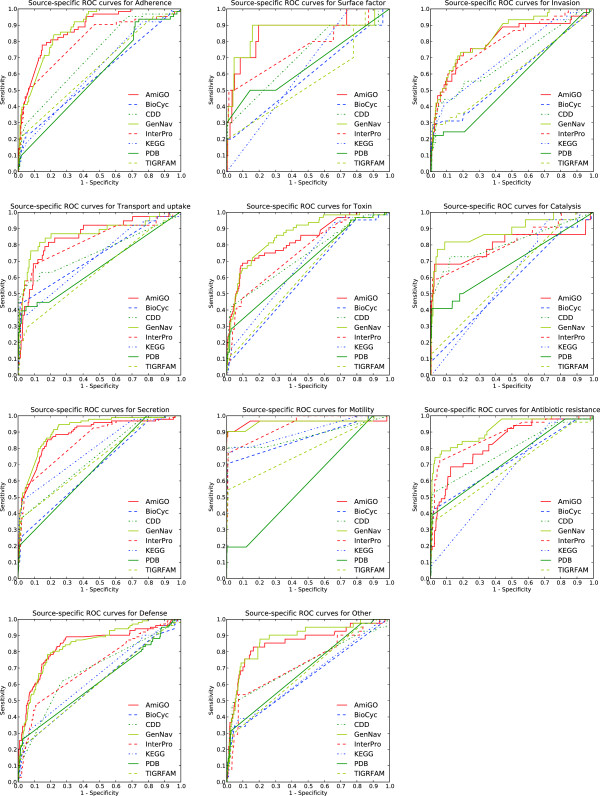
**Optimized ROCs.** ROC curves for data-integrated sources using optimized parameters.

Query graphs were generated for all 2055 proteins in the generalized virulence data set with the schema in Figure 2 using the path-based query approach described earlier. Analysis of the data focused on evaluation of performance via area under the receiver operating characteristic curve, or AUC. Three learning algorithms were tested to evaluate whether an integrated query approach can be robustly applied to different classifiers: *k* nearest-neighbor (*k*NN), ridge regression and SVMs [44-46]. The above are discriminative methods that have been successfully applied to noisy biological datasets in the past for classification problems, and we refer the reader to the above citations for the mathematical details of each approach. Briefly, a *k*NN model makes few assumptions regarding the structure of the data, and the class for an unknown instance is learned directly from the training examples via some distance metric, such that $\left(\hat{y_{i}}=\frac{1}{k}\underset{j\in N_{i}^{\left(k\right)}}{\sum }y_{j}\right)$, where members of $\left(N_{i}^{\left(k\right)}\right)$ are dictated by some distance function (e.g., in the case of (5), this distance function returned an e-value). Classifiers based on ridge regression techniques attempt linear separability of the data by obtaining the **w** that minimizes the function ∑_*i*_(*y*_*i*_−**w**^*T*^**x**_*i*_)^2^ + *λ*||**w**||^2^. In this formulation, **w** defines the class-discriminating boundary. In the SVM the function to minimize is (||**w**||^2^/2) + *C *∑_*i*_*ζ*_*i*_ s.t. (*y*_*i*_−**w**^*T*^**x**_*i*_) ≥ 1 −* ζ*_*i*_, where *C* represents a cost parameter for the “slack” variable *ζ*. While ridge regression and SVM appear similar, the regularizing functions of ridge regression (square loss) and the SVM differ (hinge loss) differ. Moreover, in the SVM *ζ* permits the presence of an optimal boundary that may not separate two classes. Both methods, however, can be formulated in an optimizable dual form, and all three can take advantage of a kernelization function, where the data points are transformed to a space that allows linear separability for otherwise non-linear data. We rely on the kernelized (non-linear) implementations provided in a open source machine learning package [47], for all experiments; appropriate free parameters were optimized for each method using grid searches.

For the general virulence dataset, we compared the above classifiers, trained on inputs generated using integrated query graphs, against the bi-layer cascaded SVM approach originally employed by Garg and Gupta (VirulentPred, [14]). VirulentPred relied on amino acid frequencies, sliding window peptide *n*-grams and a feature set derived from position-specific scoring matrices generated from PSI-BLAST searches against NBCI’s non-redundant database. Likewise, we included for comparison baseline approaches against which VirulentPred was evaluated; namely, single SVMs trained against amino acid frequencies. In this case, 1-, 2- and 3-mer frequencies of proteins were used as the feature to an SVM classifier, resulting in trainable feature spaces of size 20, 20^2^ and 20^3^, respectively.

### Specific virulence prediction evaluation procedure

Unlike the prediction of generalized virulence in the previous section, the problem of specific virulence is multiclass. Each protein was permitted to have multiple virulence labels attached to it, and thus for each classification method and source 11 different SVMs were tested in a one-versus-rest fashion. That is, each virulence category was set as the positive set of interest, and all other proteins (non-virulent proteins and virulent proteins of a differing classification) were treated as the negative set. Two primary experiments were conducted on the specific virulence set.

First, similarly to how evaluation was conducted for generalized virulence, the dataset for specific virulence was split into training and testing components; 80% of the dataset was used for training the various parameters for the classifiers and 20% retained for final testing. For the integrated query graphs, data was generated as in the generalized virulence experiment, with the same data sources and identical schema. As the SVM performed the best overall in the generalized virulence experiment (see Results), this kernel was chosen for the specific virulence classification experiment. Optimal parameters for the integrated query graph were determined via a grid search using the same procedure as generalized virulence. The parameters selected for each source and for each virulence class were those that provided the best AUC performance.

One step utilized in specific virulence that was not done in general virulence was feature selection on the integrated query graphs for the train-test split experiment, in the form of F- scores. This metric, calculated prior to SVM training and testing, provides a rough estimate of the predictive value of a feature, independent of the other features, for any given class. The calculation used here follows the formulation outlined in [48]. Let *i* correspond to the *i*^*th*^ feature in a data source. Then:

(4) $$F(i)\;=\;\frac{{\left({\bar{x}}_{i}^{\left(+\right)}\;-\;{\bar{x}}_{i}\right)}^{2}\;\;+\;\;{\left({\bar{x}}_{i}^{\left(-\right)}\;-\;{\bar{x}}_{i}\right)}^{2}}{\frac{1}{|x^{\left(+\right)}|-1}\overset{\left|x^{\left(+\right)}\right|}{\underset{k=1}{\sum }}\;\;\left(x_{k,i}^{\left(+\right)}\;-\;{\bar{x}}_{i}^{\left(+\right)}\right)\;\;+\;\;\frac{1}{|x^{\left(-\right)}|-1}\overset{\left|x^{\left(-\right)}\right|}{\underset{k=1}{\sum }}\;\;\left(x_{k,i}^{\left(-\right)}\;-\;{\bar{x}}_{i}^{\left(-\right)}\right)\;\;},$$

where, respectively, **x**^(+) ^and **x**^(−)^ are the positive and negative datasets, ${\bar{x}}^{\left(+\right)}$, ${\bar{x}}^{\left(-\right)}$, ${\bar{x}}_{i}$ are the averages of the positive, negative and complete sets of the *i*th feature, and $\left(x_{k,i}^{\left(+\right)}\right)$, $\left(x_{k,i}^{\left(-\right)}\right)$ are the values of the *i*^*th *^feature of the *k*^*th *^instance of the positive and negative sets. Features whose F-scores were in the top 25%, 50% and 75% were tested, per source in the training set. As with the SVM parameters, the features that yielded the best AUC in training were the features then used for the final test results.

The second experiment involved running six five-fold cross-validations for each class and method with the intention of obtaining measures of variance and deviation for each classifier. For each cross-validation run, the five-fold splits were the same across all classifiers to accommodate direct, paired comparison. In the case of SVM-based baseline methods and sources, Gaussian kernels were used, as they seemed to have the most consistent performance in generalized virulence prediction (see Results); parameters for the kernel and SVM were default and non-optimized, per libSVM [49]. Because 30 individual values are reported for each classifier per virulence class, paired two-tailed *t*-tests were used to measure the significance of any mean differences between the sources, and between the sources and baseline methods; *p*-values were adjusted for multiple pair-wise comparisons via Bonferroni correction.

To compare the performance of our methods against the problem of specific virulence, we used several classifiers to establish baselines. The first baseline approach was carried over from the methods used for comparing generalized virulence, and was simply a 3-mer sliding window of amino acid frequency counts. The second baseline classifier was a nearest-neighbor sequence-similarity-based approach, where a BLAST database of the specific virulence dataset was created, and classification decisions were based on mutual BLAST results of the dataset proteins against each other. Each individual protein *i* was queried against the created BLAST database, and its affinity *p* to any given class *C* was determined by:

(5) $$p_{i\in L}=\frac{\underset{n\in N_{i}^{\left(k\right)}}{\sum }1_{n}(L)}{\left|N_{i}^{\left(k\right)}\right|},$$

where the set $\left(N_{i}^{\left(k\right)}\right)$ denotes the neighborhood of *k*-nearest proteins to *i* (as determined by highest results from BLAST), and **1**_*n*_(*L*) is the indicator function, which is equal to 1 if *n *∈* L *and 0 otherwise; thus, *p* is the fraction of the *k*-nearest neighbors of *i* that have membership in *L*. This approach was used since each protein in the dataset could take on multiple classes at once and the formulation in (5) permits the measurement of membership strength for any arbitrary class, given some protein; for present purposes, the cluster size was chosen to be *k *= 3. The motivation behind this very simple approach is to measure annotation based on data from a single source, and in such a way as to emulate how an annotator may scan the best-scoring BLAST hits of a sequence to determine function [50].

The third and final baseline classifier used was also based on BLAST, but relies on using SVMs trained on pair-wise hits (with a high e-value threshold) against a BLAST database of the training set. To generate features for this third baseline classifier, each test sequence was queried against the trained BLAST database, resulting in a vector representation of a sequence’s negative log transformed e-value score to the other sequences within the database. This method is referred to as BLAST+SVM and has been used in prior experiments by others, where SVMs based on pair-wise BLAST queries outperformed or were comparable to other methods such as SVM-Fisher, SAM, PSI-BLAST, Smith-Waterman and motifs with SVMs in detecting sequences that were remotely homologous [51,52].

## Results and discussion

### General virulence

#### Comparison of data sources for predicting virulence

Table 3 shows the results of using different data sources extracted from the query graph for predicting virulence. One emergent pattern from the results was that the more coverage a data source provided, the better it performed. The notable exception is the difference between AmiGO and GenNav - both sources use GO terms linked from other sources, and have the same coverage. However, GenNav links to the parents of the GO terms, and the parents of those GO terms and so on, up to the top-level of the GO hierarchy. Despite the similarity in coverage, GenNav outperforms AmiGO by as much as 0.05. GenNav generates more data than AmiGO via self-reference, and the performance difference suggests that leveraging the ancestry of a GO term may be more useful for predictive purposes than just the immediate GO term by itself. Overall, results imply that the sources oriented around GO terms were the best performing, while TIGRFAM and BioCyc were the least predictive.

**Table 3:** Comparison of generalized classification across sources

***Data source***	***Classification method***		
	**SVM**_**(RBF)**_	**Ridge regr.**	***k*NN**
AmiGO	0.894	**0.907**	0.867
BioCyc	**0.698**	0.687	0.679
CDD	0.729	**0.760**	0.755
GenNav	**0.940**	0.935	0.878
InterPro	**0.846**	0.804	0.832
Kegg	0.733	0.778	**0.779**
Kegg (pathways)	**0.740**	0.739	0.717
Pdb	**0.740**	0.737	0.710
TigrFam	0.688	0.702	**0.704**

Results by source and method for predicting virulent and non-virulent bacterial proteins given AUC. The best performer, GenNav was run with a Gaussian kernel whose *σ *= 1.0 and regularization cost *C *= 1.0. For each method, the best performing classification approach is bolded.

To determine if the pattern carried over when empty query graphs (i.e., cases where no information for a protein was retrieved) were excluded, the same train-test process was re-ran as before, omitting any query graph from training or testing that did not yield any query results. As the SVM approach seemed to do the best on average, that statistical learning approach was used for this follow-up experiment, and the appropriate parameters were optimized for this subset of the training-testing data. Omitting empty graphs reduced the number of training and testing instances for each source, in some cases by more than 50%. However, the result was a rough sense of the predictive ability of each source, given records existed for that source in the query graph (see Table 4). Though AmiGO and GenNav maintained essentially the same scores, the rest of the sources experienced noticeable increases. Despite this overall improvement, the relative ranking of the sources remained the same, again with AmiGO and GenNav outperforming other sources.

**Table 4:** SVM classification using different data sources

***Data source***	***AUC***
AmiGO	0.886
BioCyc	0.807
CDD	0.876
GenNav	0.940
InterPro	0.883
Kegg	0.795
Kegg (pathways)	0.815
Pdb	0.875
TigrFam	0.872

Above are results by source, when empty graphs (query graphs with no returned results) are excluded from training and testing; the scores are thus those of each source given data from that source was available.

#### Comparison with competing methods for predicting virulence

Comparing the AUCs and accuracies of using weighted and integrated queries with the cascaded SVM approach, there is a marked improvement in performance. Using the best-scoring single source (GenNav), the three learning approaches were compared the amino acid frequency baseline and VirulentPred (see Table 5). Regardless of the statistical learning method used, GenNav integrated queries resulted in AUCs of 0.07-0.08 higher than the cascaded SVM approach, and approximately 0.15 greater than the sequence baseline. Accuracies are less one-sided, and in fact the *k*NN approach did only 0.053 better than the sequence baseline, suggestive of the significant amount of noise present in the retrieved data.

**Table 5:** Learned query graph performance using the VirulentPred test set

***Data src.***	***Class. method***	**AUC**	**Accuracy**
1-*mer*	SVM_(RBF)_	0.786	0.710
–	VirulentPred	0.860	0.818
GenNav	SVM_(RBF)_	0.940	0.868
GenNav	Ridge regression	0.935	0.863
GenNav	*k*NN	0.935	0.763

Comparison of the top-performing integrated predictor against a sequence baseline and VirulentPred; all methods outlined in the table used the same set of proteins.

### Specific virulence

#### Source-against-source performance

Across all specific virulence categories the AUCs of the GenNav and AmiGO data sources, whose records were indirectly queried from the seeding protein, outperformed all other methods and data sources, in some cases by very large margins (see Table 6 for the feature spaces for each source). Comparisons of the GO-based results to the other sources are further indicative that a learner based on integrated queries provides a better classifier. Notably, two other sources that are more lightly integrated, KEGG and InterPro also perform well relative to the other sources; under Kendall’s rank correlation, coverage (as defined, per source, by the fraction of graphs with one or more records present in a query graph) was significantly related to AUC.

**Table 6:** Feature space sizes across sources

***Data src.***	***Num. features***	***Feature type***
AmiGO	5102	terms
BioCyc	1674	proteins, pathways
Cdd	6463	models
GenNav	6425	terms
InterPro	3540	models
Kegg	234	pathways
Pdb	7954	structures
TigrFam	1109	models

Number of features per source used for specific virulence predictions. Individual source feature sizes are reported before any feature selection.

Further analysis of the ROC scores reveals other interesting results. Category 8, Motility, was relatively trivial to classify not merely by GenNav but by other sources as well, including KEGG and InterPro. One explanation for these results was that the motility of bacterial pathogens, and indeed bacteria in general, is a very well characterized process, and proteins related to bacterial motion are well-annotated and unambiguous. Despite its coverage in comparison to sources like TIGRFAM and BioCyc, and contrary to the case in other categories, PDB records failed to predict motility well. This may partly be due to the fact that motility-related proteins, given their high probability of containing transmembrane regions, are difficult to structurally elucidate and thus good exemplars of this class are more absent in this database.

#### Integrated query graph learning versus baseline methods

**Figure 3:**
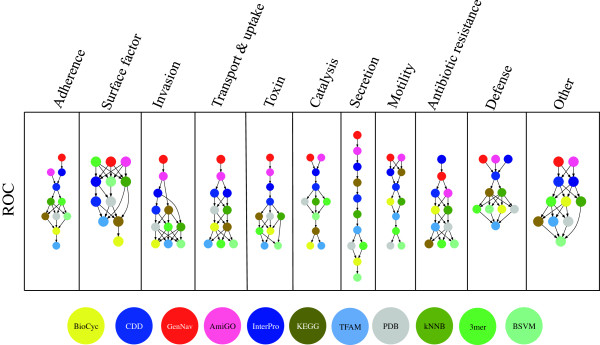
**Specific ROC comparisons.** Statistical significances based on six five-fold cross-validations for all 11 virulence classes. An arrow from a head source or method to a tail source or method transitively indicates better pairwise performance from the head against the tail. Results, via two- tailed t-test, for area under the ROC are shown and nodes are color-coded based on source type (blue indicates domain- or motif-based sources, red GO term-based sources, yellow- brown for pathways, gray for structural sources and green for baseline methods).

Besides making inter-source comparisons, we compared our query-based learning methods to baseline methods. Figure 3 shows the pair-wise comparison results of six five-fold cross validation runs with the sources and baseline methods, with better methods appearing higher in the graph. Note that unlike the parameter-optimized results in the previous section, feature selection based on training data for the sources was not performed and classification was done using an unpruned feature set.

Statistical significance of pairwise comparisons are also visible in Figure 3 via transitive arrows. It stands out that in all but one virulence class, at least one of the queried data sources outperforms all baseline methods; 3-mer performance on the Surface factor label was exemplary compared to most sources and the other baseline methods (see Additional file 3 for exact statistical results). However, it was also the label with the fewest instances. In eight of the virulence classes, the ROC curves of GO-term based methods outperform not just baseline methods, but all other sources as well. Interestingly, for proteins related to antibiotic resistance, such as drug efflux pumps, InterPro does significantly better than all other sources and methods.

**Figure 4:**
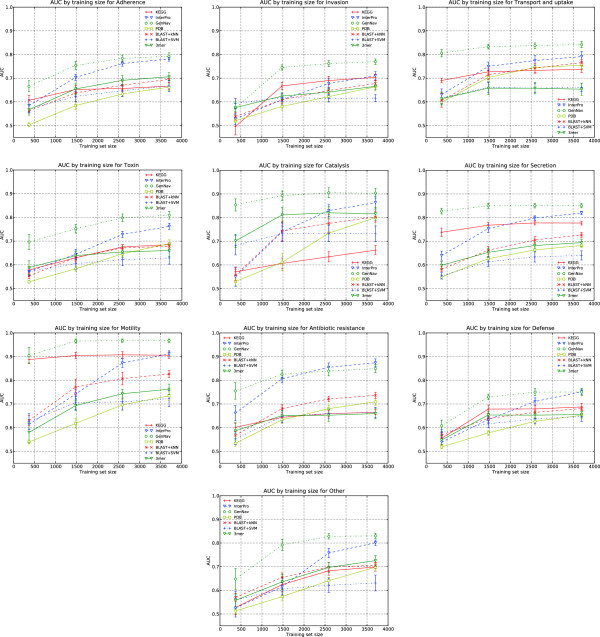
**Performance as an effect of dataset size.** Average AUCs and 95% confidence intervals for a subset of sources and the baselines by training set size, based on three five-fold cross-validations. The ‘Surface factor’ virulence class is omitted due to the small number of instances present in the training set.

It was also important to determine how well virulence classes could be discerned using varying levels of training set sizes. The motivation behind measuring this was to gauge how well known a family of virulence factors may need to be for successful identification ‘in the wild.’ Figure 4 displays the AUCs of a subset of sources (those that performed best in the generalized virulence classification test) and all baseline methods from three paired five-fold cross-validations under different and increasing training set sizes -10%, 40%, 70% and 100% of the original training set sizes; testing sets remain untouched.

These findings strongly illustrate that the performance of some classifiers do not significantly change with the number of instances seen. In the case of the Motility and Secretion classes, both KEGG and GenNav perform essentially the same and with little change, though for other sources such as InterPro the AUC increased with the training set size. This led to the conclusion that some data sources may better characterize classes than other sources, and that in the case of GenNav and KEGG for Secretion, there are likely a set of terms or pathways that commonly describe pathogen secretory mechanisms, and that these annotations are widespread across the set of secretion-related proteins. Also of note was the performance of GenNav under small training set size conditions. Other sources and methods generally tend to perform poorly (< 0.7 AUC) with training sets less than 500 instances, whereas GenNav does considerably better in 7 of the 10 cases often by more than 0.1. This suggests that heavily integrated sources, such as GenNav, may have additional utility over other methods when the number of seen and known instances from which to train are very low, leading to the hypothesis that integrated methods may do better under more ambiguous conditions when compared to competing approaches.

#### Estimating the advantage of multiple features for specific virulence

Thus far, results have strongly suggested that some categories are much easier to classify than others. For example, Motility was easily predicted across all integrated sources, and for some even at very low numbers of training instances. In comparison, other virulence classes such as Invasion and Defense remain harder to identify with strong confidence. Because many of these nuances in performance are both source- and label-specific, it was of interest to generate ROCs without a classifier, and using only the weights determined from the propagation algorithm in the query graphs. For each category and source, the F-score per (5) was computed, and the highest-performing feature was kept. Recall that each feature was assigned a weight from the query graph; this value was used as the thresholding function for the generation of AUC scores. The result of this was essentially a very basic classifier, *Top-F1*, which relied only on the single-most discriminating feature of each source for each label. The AUC for this classifier represents predictions ignorant of any value in combining multiple features; comparisons of this with other methods would thus illustrate any advantages or disadvantages from using more sophisticated approaches on the query graph data.

**Figure 5:**
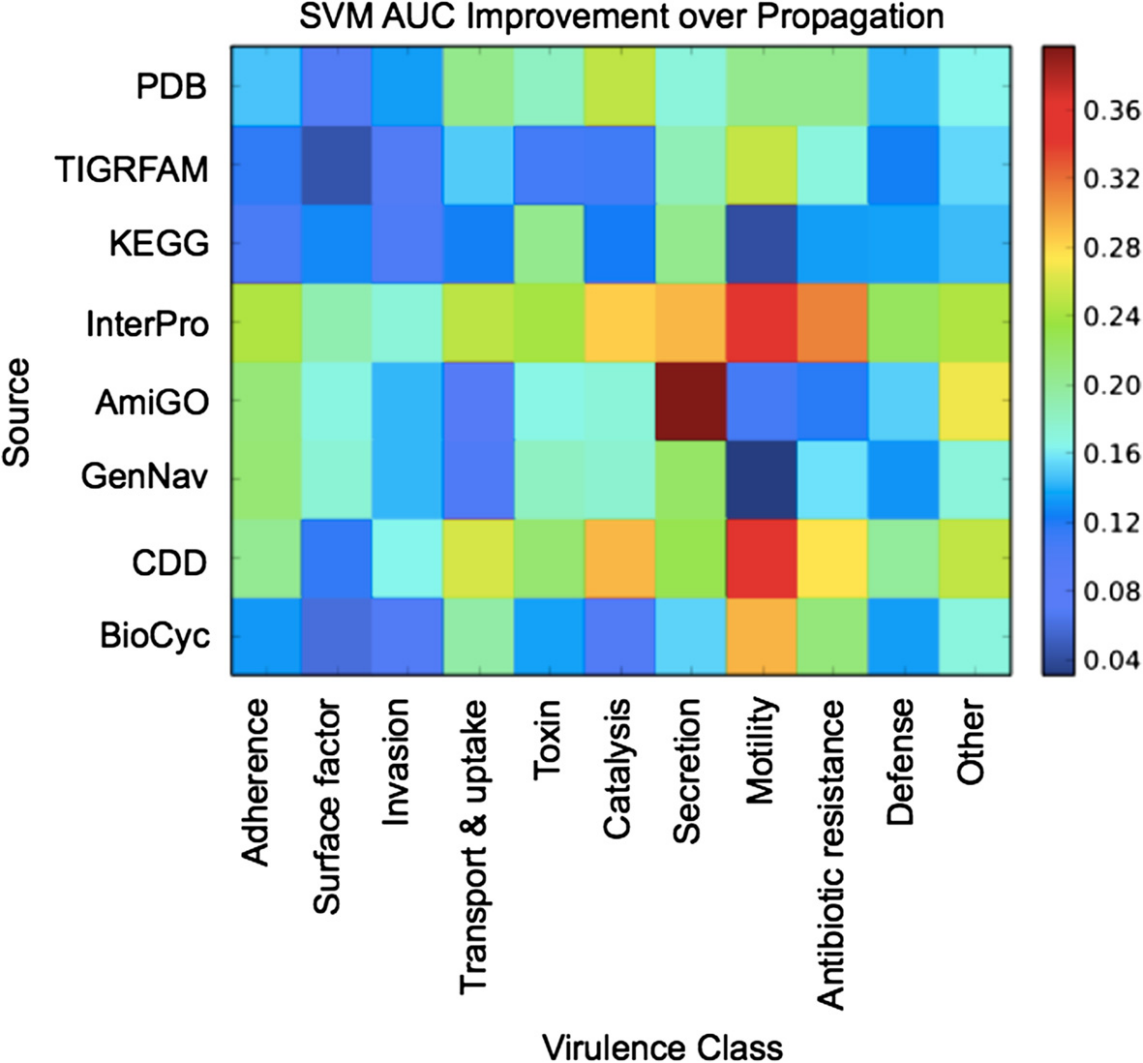
**Improvement over naïve prediction.** Improvement in AUC of using an SVM for classification for cross-validated tests over *Top*−*F*1. The colors in each cell correspond to the difference in AUC between using an SVM for prediction and using the top-performing feature per source, per label. Higher values indicate cases where there is added benefit in considering multiple features via SVMs.

Figure 5 shows a heatmap of the difference between SVM AUCs. Across all sources and labels, the ROC curves using SVM-based methods demonstrated added utility over *Top-F1*. Colors trending toward the deep blue end of the spectrum represent modest increases in performance, while colors closer to deep red are more marked improvements for the SVM method over *Top-F1* It was clear that there is marginal benefit to using a more sophisticated classification method for KEGG and GenNav on the Motility class, although other sources such as BioCyc derive a noticeable advantage from using SVMs for classification; for some sources and labels the mere presence of a single feature can be strongly indicative of membership.

At the same time, other sources benefit greatly from the combination of features, namely InterPro and CDD, the two sources that on average have the highest improvement of using SVMs over *Top-F1*. At the opposite end, TIGRFAM and BioCyc show the least overall improvement, with the GO-term based sources (AmiGO, GenNav) showing moderate improvement. Examining the GO-term based sources shows interesting differences between using only direct annotation information (AmiGO) and enrichment via traversing the GO hierarchy (GenNav). While most of the changes between the two sources are commensurate, AmiGO strongly benefits from the use of SVMs for the Secretion category. One conclusion from this is that there are several top-level terms in GO that suggest secretion, and that under GenNav these terms are retrieved; under AmiGO, however, this information was not available, but was ameliorated by the availability of terms that may share mutual parents.

## Conclusions

In the case of general virulence, and in the majority of specific virulence classes, a classification approach that used integrated queries as input performed significantly better than the baselines and competing approaches. Surprisingly, it was found that the data sources several nodes away from our query were the most predictive, which may at first glance seem counterintuitive. GenNav and AmiGO both are sources indirectly connected to the initial queries in the query graph, yet they were the best performing. A large part of this may be due to their superior coverage, as they are referenced by other data sources, but even when empty graphs are omitted it was found that these data sources still out-scored the other sources directly connected to the query. This reinforces the position that, as it pertains to using biological databases for classifying protein data, shallow queries are generally not sufficient. Manually, this information can be difficult to sift through, and using robust methods to cut through the “chaff” is indispensable. This is particularly salient given the results that learning methods more resistant to noise were the best at identifying virulent proteins.

A limitation that is not explored in this work but is self-evident in implementation is that performance will likely be very dependent on the choice of data sources and cross- linkages. Moreover, as data sources evolve, are expanded or curated, source-specific findings may vary. This caveat is particularly applicable to sources which are directly queried (e.g., CDD); indirect features (e.g., GO terms) may be more robust to this effect as multiple sources are integrated. In either case, query proteins ideally will have some limited sequence similarity to other proteins; truly novel proteins may not provide sufficient results for classification. In such instances, relaxing stringency to provide results with very low homology may be effective, though accuracy and interpretability will likely suffer. Another notable limitation involves evaluating the classifications. The circuitous nature of sequence annotation from biological databases makes it difficult to identify annotations that were derived transitively. While we attempted to address this problem by omitting from the query graph any results with 100% similarity to the query sequences and disallowed sequence results from serving as seeds for other sequence queries, it is likely that protein families or domains with GO assignments, which may have been curated based in part on the query sequence, were returned and used for classification. Based on our analysis of the results, however, we believe the most influential reason for the dominating performance of GO terms may be how informative the features are relative to other sources. Recall that the AUC findings between AmiGO (linked GO terms only) and GenNav (linked and hierarchical GO terms) was greater than the difference between AmiGO and the top-performing non-GO sources (Table 4); indeed, InterPro performs similarly to linked GO, but does not outperform hierarchical GO. This leads us to believe that there is inherent value in using a standardized and rich vocabulary for classification beyond coverage alone. We find that there is value in cross-linking across data sources, and that while provenance and quality of data are naturally very important even the most naive retrieval approaches can provide useful information on identifying protein virulence, and perhaps protein class in general.

Notably, our method used each data source, weighted via query integration, to serve as separate inputs to individual classifiers. This approach resulted in multiple source-specific classifiers that only utilized a subgraph of the entire query graph. A perhaps preferable alternative would be single classifier that utilized the entire graph. To that end, we explored additional methods of source integration to augment query weighting, and in particular kernel integration, where kernels generated for each source were additively combined before learning. Our initial findings using this approach resulted in marginal improvement using naïve equal kernel weighting, at prohibitively high computational cost. Achieving full query graph integration for the classification phase is a reasonable extension of this work, however, and further work is needed to explore ways of optimizing kernel weight selection to improve performance and justify the cost [53].

By presenting a method of exploting this data in the context of virulence and presenting the results, a major insight is that even without extensive manual curation integrated data can be very effective for prioritization of both virulence factors and general function prediction. This approach scales well against both the number of sources incorporated and the amount of ground truth information known, making it an appropriate choice for high-throughput biological research. Towards this end, our methods have been incorporated into the target selection pipeline of the Seattle Structural Genomics Center for Infectious Disease (SSGCID) for down-selecting virulence related proteins for structural elucidation [54]. Lastly, results produced by this research are possible targets of health-related interest in public health and infectious disease biology; highlighting these proteins for 5itional study may further improve knowledge of pathogenesis and disease.

## Competing interests

The authors declare they have no competing interests.

## Authors’ contributions

EC, PTH and PJM conceived and refined the methods and study design, drafted the manuscript and reviewed the results. EC wrote the related software and conducted the analysis. Work was performed while EC was affiliated with the University of Washington, Seattle Biomedical Research Institute and Lawrence Livermore National Laboratory. All authors read and approved the final manuscript.

## Supplementary Material

Additional file 1 **faa — Specific virulence protein sequences.** This FASTA file contains the 3700 protein sequences used for training and testing the specific virulence classifiers. Note that the FASTA sequence headers contain only the unique identifiers; labels are located in a separate file. Click here for file

Additional file 2 **txt – Specific virulence class labels.** This tab-delimited file contains two columns. The first column is the unique sequence identifier matched to some protein sequence in the specific virulence FASTA file, and the second column indicates the specific virulence label, per Table 2. Non-virulent proteins are assigned class ‘12’ in this file. Click here for file

Additional file 3 **pdf — Statistical significance outcomes of method and source comparisons.** A PDF containing tabular results of statistical significance testing from six five-fold cross-validations of integrated data against each other and baselines. Data from these tables was used to construct the comparison networks in Figure 3. Click here for file
