# Supplementary material for: Learning virulent proteins from integrated query networks
Source: BMC Bioinformatics. 2012 Dec 2;13:321. doi: 10.1186/1471-2105-13-321 (PMC3560104; doi:10.1186/1471-2105-13-321)
Supplement: Additional file 3 — pdf — Statistical significance outcomes of method and source comparisons. A PDF containing tabular results of statistical significance testing from six five-fold cross-validations of integrated data against each other and baselines. Data from these tables was used to construct the comparison networks in Figure 3. [file 1471-2105-13-321-S3.pdf]

Learning pathogenic proteins from integrated query networks: Supplemental Tables

Eithon Cadag Peter Tarczy-Hornoch Peter J. Myler

Tables 1 to 11 contain the statistical significance tests for the six five-fold cross-validations of the integrated data sources against the baseline, according to the results of paired t-tests. Each cell contains a Bonferroni-adjusted p-value if the source or method describing the row statistically outperforms the source or method on the column, a (+) if the source along the row is not statistically better than the source or method along the column and a (-) if the source or method along the row is the same or worse (statistically or otherwise). Significance for these comparisons was set at $\alpha = 0.05$.

For these tables, the sources are listed as BioCyc (BCyc), CDD, GenNav (GN), AmiGO (AG), InterPro (IPro), KEGG, TIGRFAM (TFAM), PDB, BLAST with kNN (kNNB), 3mers frequencies and BLAST with SVM (BSVM).

	BCyc	CDD	GN	AG	IPro	KEGG	TFAM	PDB	kNNB	3mer	BSVM
BioC	-	-	-	-	-	-	6.06e-05	-	-	-	-
CDD	1.86e-04	-	-	-	-	+	1.13e-10	9.68e-05	-	-	-
GN	0.00e+00	0.00e+00	-	+	5.85e-04	0.00e+00	0.00e+00	0.00e+00	4.13e-11	1.60e-08	0.00e+00
AG	0.00e+00	0.00e+00	-	-	5.51e-03	0.00e+00	0.00e+00	0.00e+00	8.16e-10	1.02e-06	0.00e+00
IPro	0.00e+00	0.00e+00	-	-	-	0.00e+00	0.00e+00	0.00e+00	3.76e-05	4.99e-04	3.44e-12
KEGG	+	-	-	-	-	-	3.90e-08	+	-	-	-
TFAM	-	-	-	-	-	-	-	-	-	-	-
PDB	+	-	-	-	-	-	3.17e-05	-	-	-	-
kNNB	5.15e-11	5.96e-08	-	-	-	4.18e-10	3.41e-11	3.48e-09	-	-	1.39e-02
3mer	7.45e-13	4.56e-11	-	-	-	8.55e-13	0.00e+00	3.66e-13	+	-	7.07e-08
BSVM	4.47e-07	4.87e-03	-	-	-	2.11e-05	2.44e-14	1.07e-05	-	-	-

Table 1: ROC p-values for the Adherence virulent class.

	BCyc	CDD	GN	AG	IPro	KEGG	TFAM	PDB	kNNB	3mer	BSVM
BioC	-	-	-	-	-	-	-	-	-	-	-
CDD	1.13e-09	-	-	-	-	4.05e-04	3.20e-08	3.23e-04	-	-	-
GN	0.00e+00	4.94e-10	-	+	2.62e-07	1.22e-14	0.00e+00	3.43e-12	1.31e-04	-	6.94e-04
AG	0.00e+00	1.57e-09	-	-	4.28e-06	1.22e-14	0.00e+00	5.78e-12	2.41e-05	-	3.02e-03
IPro	3.91e-13	3.14e-03	-	-	-	1.52e-07	7.36e-12	1.14e-05	+	-	-
KEGG	3.26e-02	-	-	-	-	-	+	-	-	-	-
TFAM	+	-	-	-	-	-	-	-	-	-	-
PDB	1.93e-05	-	-	-	-	+	3.54e-03	-	-	-	-
kNNB	2.57e-07	+	-	-	-	5.67e-05	2.94e-05	+	-	-	-
3mer	0.00e+00	3.60e-11	+	+	1.77e-07	0.00e+00	0.00e+00	4.27e-13	1.12e-05	-	3.03e-05
BSVM	1.44e-10	7.06e-03	-	-	+	9.75e-08	3.15e-09	6.91e-05	+	-	-

Table 2: ROC p-values for the Surface factor virulent class.

	BCyc	CDD	GN	AG	IPro	KEGG	TFAM	PDB	kNNB	3mer	BSVM
BioC	-	-	-	-	-	-	-	-	-	-	-
CDD	4.53e-06	-	-	-	-	-	4.11e-06	1.50e-02	-	-	6.24e-03
GN	0.00e+00	8.06e-13	-	3.73e-06	6.25e-09	1.22e-14	0.00e+00	0.00e+00	1.41e-11	1.16e-09	0.00e+00
AG	3.05e-13	2.41e-10	-	-	4.66e-05	2.69e-09	1.22e-14	8.55e-14	8.43e-09	4.25e-07	2.93e-13
IPro	2.73e-11	1.83e-05	-	-	-	+	1.95e-12	1.24e-08	+	+	1.28e-09
KEGG	7.80e-07	+	-	-	-	-	2.47e-07	9.78e-04	+	+	1.47e-06
TFAM	+	-	-	-	-	-	-	-	-	-	-
PDB	+	-	-	-	-	-	+	-	-	-	+
kNNB	6.40e-06	+	-	-	-	-	1.11e-05	1.94e-04	-	+	4.25e-04
3mer	2.58e-04	+	-	-	-	-	2.40e-05	4.82e-03	-	-	5.64e-08
BSVM	+	-	-	-	-	-	+	-	-	-	-

Table 3: ROC p-values for the Invasion virulent class.

	BCyc	CDD	GN	AG	IPro	KEGG	TFAM	PDB	kNNB	3mer	BSVM
BioC	-	-	-	-	-	-	7.53e-08	-	-	3.54e-04	3.10e-03
CDD	6.11e-13	-	-	-	+	6.70e-07	1.22e-14	4.55e-03	+	5.88e-11	3.09e-10
GN	0.00e+00	8.10e-08	-	7.16e-05	7.90e-08	1.22e-14	0.00e+00	2.55e-10	4.34e-06	0.00e+00	0.00e+00
AG	1.28e-12	4.51e-04	-	-	2.41e-04	4.36e-12	0.00e+00	4.49e-08	2.67e-03	0.00e+00	0.00e+00
IPro	8.08e-08	-	-	-	-	1.06e-06	0.00e+00	+	+	9.51e-12	6.01e-10
KEGG	+	-	-	-	-	-	1.16e-07	-	-	9.14e-08	9.24e-05
TFAM	-	-	-	-	-	-	-	-	-	-	-
PDB	4.07e-05	-	-	-	-	1.70e-02	1.18e-11	-	-	4.65e-08	2.65e-07
kNNB	6.72e-04	-	-	-	-	3.86e-05	1.01e-09	+	-	3.04e-11	8.20e-10
3mer	-	-	-	-	-	-	+	-	-	-	-
BSVM	-	-	-	-	-	-	+	-	-	+	-

Table 4: ROC p-values for the Transport and uptake virulent class.

	BCyc	CDD	GN	AG	IPro	KEGG	TFAM	PDB	kNNB	3mer	BSVM
BioC	-	-	-	-	-	-	9.20e-04	-	-	-	+
CDD	2.61e-06	-	-	-	-	+	1.95e-13	+	+	6.55e-03	3.16e-05
GN	0.00e+00	6.11e-14	-	1.10e-02	8.04e-04	0.00e+00	0.00e+00	1.34e-13	1.22e-14	0.00e+00	0.00e+00
AG	0.00e+00	4.52e-13	-	-	4.35e-02	0.00e+00	0.00e+00	4.15e-13	0.00e+00	0.00e+00	0.00e+00
IPro	1.22e-14	2.30e-11	-	-	-	2.30e-12	0.00e+00	1.45e-10	9.67e-11	4.27e-13	1.22e-13
KEGG	4.00e-03	-	-	-	-	-	2.52e-10	-	+	+	6.69e-04
TFAM	-	-	-	-	-	-	-	-	-	-	-
PDB	3.59e-04	-	-	-	-	+	4.45e-10	-	+	2.48e-02	5.48e-05
kNNB	+	-	-	-	-	-	1.41e-03	-	-	+	8.94e-04
3mer	+	-	-	-	-	-	2.84e-02	-	-	-	+
BSVM	-	-	-	-	-	-	+	-	-	-	-

Table 5: ROC p-values for the Toxin virulent class.

	BCyc	CDD	GN	AG	IPro	KEGG	TFAM	PDB	kNNB	3mer	BSVM
BioC	-	-	-	-	-	-	-	-	-	-	-
CDD	0.00e+00	-	-	-	-	7.62e-08	9.77e-14	-	-	-	-
GN	0.00e+00	8.94e-12	-	+	2.40e-04	0.00e+00	0.00e+00	1.44e-11	1.44e-04	3.59e-06	3.88e-08
AG	0.00e+00	1.01e-11	-	-	6.74e-04	0.00e+00	0.00e+00	2.62e-11	4.48e-04	3.47e-07	1.61e-07
IPro	0.00e+00	3.23e-10	-	-	-	0.00e+00	0.00e+00	3.98e-09	+	+	9.23e-08
KEGG	1.92e-04	-	-	-	-	-	+	-	-	-	-
TFAM	+	-	-	-	-	-	-	-	-	-	-
PDB	3.66e-14	+	-	-	-	1.11e-09	9.40e-13	-	-	-	+
kNNB	0.00e+00	1.77e-04	-	-	-	0.00e+00	0.00e+00	2.06e-02	-	-	1.26e-02
3mer	0.00e+00	2.04e-06	-	-	-	1.22e-14	1.22e-14	6.69e-04	+	-	1.91e-03
BSVM	8.43e-13	+	-	-	-	6.94e-07	9.18e-11	-	-	-	-

Table 6: ROC p-values for the Catalysis virulent class.

	BCyc	CDD	GN	AG	IPro	KEGG	TFAM	PDB	kNNB	3mer	BSVM
BioC	-	-	-	-	-	-	-	-	-	-	+
CDD	0.00e+00	-	-	-	-	-	9.55e-08	1.22e-13	-	+	2.95e-11
GN	0.00e+00	0.00e+00	-	7.42e-07	4.53e-10	1.21e-11	0.00e+00	0.00e+00	0.00e+00	0.00e+00	0.00e+00
AG	0.00e+00	0.00e+00	-	-	3.19e-04	7.02e-08	0.00e+00	0.00e+00	2.44e-14	0.00e+00	0.00e+00
IPro	0.00e+00	0.00e+00	-	-	-	8.97e-04	0.00e+00	0.00e+00	1.72e-09	0.00e+00	0.00e+00
KEGG	0.00e+00	7.82e-13	-	-	-	-	0.00e+00	0.00e+00	2.77e-02	7.77e-11	0.00e+00
TFAM	3.56e-05	-	-	-	-	-	-	+	-	-	2.39e-06
PDB	+	-	-	-	-	-	-	-	-	-	1.13e-04
kNNB	1.73e-11	5.98e-04	-	-	-	-	2.97e-09	3.11e-10	-	1.04e-05	0.00e+00
3mer	3.10e-10	-	-	-	-	-	2.96e-05	3.57e-07	-	-	7.69e-13
BSVM	-	-	-	-	-	-	-	-	-	-	-

Table 7: ROC p-values for the Secretion virulent class.

	BCyc	CDD	GN	AG	IPro	KEGG	TFAM	PDB	kNNB	3mer	BSVM
BioC	-	-	-	-	-	-	1.03e-05	0.00e+00	+	7.38e-10	9.24e-12
CDD	8.03e-09	-	-	-	-	-	1.87e-11	0.00e+00	1.59e-05	7.33e-14	1.22e-13
GN	1.22e-14	8.61e-12	-	+	9.46e-09	6.40e-08	0.00e+00	0.00e+00	1.22e-12	0.00e+00	0.00e+00
AG	9.77e-14	1.42e-10	-	-	3.96e-08	2.45e-07	0.00e+00	0.00e+00	1.49e-12	0.00e+00	0.00e+00
IPro	3.16e-09	3.86e-02	-	-	-	-	0.00e+00	0.00e+00	3.31e-07	0.00e+00	3.66e-14
KEGG	9.15e-07	+	-	-	+	-	1.45e-11	0.00e+00	1.48e-07	0.00e+00	3.66e-14
TFAM	-	-	-	-	-	-	-	5.52e-07	-	+	2.09e-05
PDB	-	-	-	-	-	-	-	-	-	-	+
kNNB	-	-	-	-	-	-	+	2.41e-05	-	+	8.93e-05
3mer	-	-	-	-	-	-	-	+	-	-	2.54e-02
BSVM	-	-	-	-	-	-	-	-	-	-	-

Table 8: ROC p-values for the Motility virulent class.

	BCyc	CDD	GN	AG	IPro	KEGG	TFAM	PDB	kNNB	3mer	BSVM
BioC	-	-	-	+	-	5.43e-11	+	+	7.75e-08	0.00e+00	0.00e+00
CDD	8.02e-06	-	-	3.32e-05	-	1.22e-14	4.77e-07	8.52e-11	3.64e-10	0.00e+00	0.00e+00
GN	2.73e-08	+	-	1.47e-13	-	0.00e+00	1.05e-08	1.41e-08	2.15e-12	0.00e+00	0.00e+00
AG	-	-	-	-	-	1.94e-07	+	+	3.42e-05	0.00e+00	0.00e+00
IPro	1.22e-14	1.08e-07	1.14e-04	8.55e-14	-	0.00e+00	0.00e+00	1.59e-13	1.22e-14	0.00e+00	0.00e+00
KEGG	-	-	-	-	-	-	-	-	+	3.89e-11	2.03e-12
TFAM	-	-	-	-	-	9.24e-09	-	+	7.09e-06	0.00e+00	0.00e+00
PDB	-	-	-	-	-	4.27e-06	-	-	1.78e-04	4.15e-13	3.66e-14
kNNB	-	-	-	-	-	-	-	-	-	5.92e-10	1.80e-09
3mer	-	-	-	-	-	-	-	-	-	-	1.10e-02
BSVM	-	-	-	-	-	-	-	-	-	-	-

Table 9: ROC p-values for the Antibiotic resistance virulent class.

	BCyc	CDD	GN	AG	IPro	KEGG	TFAM	PDB	kNNB	3mer	BSVM
BioC	-	-	-	-	-	-	+	-	-	-	-
CDD	2.06e-12	-	-	-	-	+	4.73e-10	7.36e-11	+	+	9.29e-04
GN	1.22e-14	7.18e-10	-	+	+	3.26e-10	0.00e+00	0.00e+00	3.47e-09	1.22e-14	7.94e-13
AG	1.22e-14	3.98e-10	-	-	+	2.60e-10	0.00e+00	0.00e+00	1.04e-08	4.03e-13	9.77e-14
IPro	1.22e-14	1.31e-09	-	-	-	5.23e-08	0.00e+00	0.00e+00	3.73e-07	4.13e-10	1.47e-11
KEGG	1.12e-05	-	-	-	-	-	5.33e-11	2.16e-07	+	+	1.02e-03
TFAM	-	-	-	-	-	-	-	-	-	-	-
PDB	+	-	-	-	-	-	+	-	-	-	-
kNNB	3.42e-02	-	-	-	-	-	1.69e-03	2.50e-02	-	+	+
3mer	4.82e-02	-	-	-	-	-	9.27e-05	1.87e-02	-	-	+
BSVM	+	-	-	-	-	-	+	+	-	-	-

Table 10: ROC p-values for the Defense virulent class.

	BCyc	CDD	GN	AG	IPro	KEGG	TFAM	PDB	kNNB	3mer	BSVM
BioC	-	-	-	-	-	+	3.26e-02	1.08e-02	+	-	8.04e-09
CDD	5.25e-06	-	-	-	-	2.29e-05	2.02e-09	3.84e-11	1.73e-08	2.96e-05	0.00e+00
GN	1.05e-12	9.44e-08	-	+	1.32e-05	2.08e-13	0.00e+00	0.00e+00	3.66e-14	1.28e-10	0.00e+00
AG	5.21e-11	8.04e-07	-	-	4.37e-04	5.32e-11	4.88e-14	1.22e-14	2.44e-14	5.69e-10	0.00e+00
IPro	6.14e-09	+	-	-	-	2.18e-06	3.59e-11	1.95e-12	8.34e-10	1.10e-06	0.00e+00
KEGG	-	-	-	-	-	-	+	+	+	-	2.72e-07
TFAM	-	-	-	-	-	-	-	+	+	-	2.00e-06
PDB	-	-	-	-	-	-	-	-	-	-	9.42e-07
kNNB	-	-	-	-	-	-	-	+	-	-	5.43e-05
3mer	+	-	-	-	-	+	+	2.73e-02	3.44e-02	-	2.84e-10
BSVM	-	-	-	-	-	-	-	-	-	-	-

Table 11: ROC p-values for the Other virulent class.
